# Pulse of Generosity: A Qualitative Insight Into the Knowledge, Attitudes, and Motivations of Voluntary Blood Donors at a Tertiary Care Hospital, Maldives

**DOI:** 10.1155/bmri/9999689

**Published:** 2026-03-09

**Authors:** Khadeeja, Aishath Selna, Adam Khaleel Yoosuf

**Affiliations:** ^1^ Department of Pathology, Indira Gandhi Memorial Hospital, Male, Maldives, igmh.gov.mv; ^2^ Institute for Research and Innovation, Villa College, Male, Maldives; ^3^ Department of Pathology, ADK Hospital, Male, Maldives, adkhospital.mv

**Keywords:** blood bank, Maldives, recruitment, retention, voluntary donors

## Abstract

**Background:**

Blood donation plays a vital role in healthcare, especially in the Maldives, where a high prevalence of thalassemia creates a significant demand for regular blood transfusions. Despite this need, the country relies heavily on directed blood donations, with limited participation from voluntary nonremunerated donors (VNRDs). Addressing donor recruitment, retention, and knowledge gaps is essential to ensure a safe and sufficient blood supply.

**Aim:**

This study is aimed at assessing the knowledge, attitudes, and motivations of voluntary blood donors registered at a tertiary care blood bank and at identifying strategies to enhance recruitment and retention of regular voluntary blood donors.

**Method:**

A qualitative approach was adopted, with data collected through interviews with 12 voluntary blood donors. Thematic analysis was conducted to explore participants′ knowledge, attitudes, and motivations and to identify challenges and potential solutions for improving voluntary blood donation rates.

**Findings:**

The study revealed that while donors displayed positive attitudes toward blood donation, misconceptions about the safest type of donors persisted, with 25% believing family donors were safer than voluntary donors. Key barriers included a lack of structured recruitment programs, limited donor retention efforts, and an insufficient focus on donor recognition. Participants recommended awareness campaigns, improved donation facilities, personalized donor engagement, and enhanced recognition programs to encourage repeat donations.

**Conclusion:**

Increasing voluntary blood donations in the Maldives requires a donor‐centric approach, incorporating structured recruitment and retention strategies, robust awareness campaigns, and improved donor experiences. These findings provide actionable insights to strengthen the national blood transfusion system and ensure a sustainable and safe blood supply.

## 1. Introduction

Blood is a critical component of modern healthcare, essential for treating various conditions, from trauma to chronic illnesses [1]. The increasing demand for blood and its products, driven by advancements in medical treatments, higher life expectancy, and a rise in conditions such as thalassemia [2], underscores the need for a reliable and safe blood supply. Studies have indicated that the demand for blood is on the rise due to factors such as aggressive surgical procedures, organ transplants [3], cancer patients, pregnant people [4], emergencies like road traffic accidents, anemia [5], and genetic disorders such as thalassemia [2]. Hence, as blood cannot be manufactured, nor is there a substitute for blood, ensuring a steady supply through donation is crucial for saving lives [6].

Globally, the challenge remains to ensure that blood supplies are sufficient and safe. While some progress has been made toward achieving 100% voluntary blood donation, the increase has been slow and uneven across countries. The WHO′s 2006 global survey revealed that only 56 out of 124 countries had made strides toward this goal, with the remaining 68 either stagnant or declining in their voluntary donation rates [7]. Moreover, from 2008 to 2018, a gradual increase of 10.7 million donations from voluntary nonremunerated donors (VNRDs) was recorded, and 79 countries collected more than 90% of their blood supply from voluntary unpaid blood donations (38 high‐income countries, 33 middle‐income countries, and eight low‐income countries). This includes 64 countries with 100% (or more than 99%) of their blood supply from voluntary unpaid blood donors. However, from 54 countries, more than 50% of the blood supply is still dependent on family/replacement and paid blood donors (8 high‐income countries, 36 middle‐income countries, and 10 low‐income countries) [8]. This situation is compounded by the challenges faced in achieving a reliable and safe blood supply while managing the quality and safety of blood products.

Maldives is among the countries that receive support from WHO, a country of approximately 1192 coral islands; the geography poses significant challenges to healthcare delivery [9], including blood transfusion services. The Maldivian Blood Services (MBS) and other hospital‐based blood banks such as those at Indira Gandhi Memorial Hospital (IGMH), ADK Hospital, Treetop Hospital (TTH), and Hulhumale′ Hospital based in the central region of Maldives as well as blood banks in the urban are crucial in providing blood components. Especially for a country like the Maldives, where there is a high prevalence of transfusion‐dependent *β*‐thalassemia cases, with approximately 28 new cases reported annually and a significant carrier rate affecting up to 18% of the population [10], the need for a consistent and safe blood supply is critical. This reliable blood supply is essential for addressing the significant burden of thalassemia and other anemia‐related conditions in the Maldives.

The proportion of voluntary nonremunerated blood donation in the Maldives ranged between 10% and 30% [11]. A decade later, this percentage remains almost unchanged, with the proportion still less than 50%, as highlighted in the WHO (2023) report, “Voluntary Non‐Remunerated Blood Donations to Ensure Blood Safety in the WHO South‐East Asia Region to Support Universal Health Coverage.” This stagnation underscores the need for renewed efforts and strategic interventions to increase VNRD in the Maldives, ensuring a safe and sustainable blood supply to support universal health coverage [12].

The National Blood Policy of 2004 and 2018 emphasizes the need for a well‐coordinated national blood transfusion service, recommends phasing out direct donors to achieve 100% voluntary blood donation, and promotes donor recruitment and retention [13]. To align with the policy, working on increasing this proportion is crucial to meeting WHO recommendations to ensure a safe and adequate blood supply. Despite global research on blood donation, there is a lack of studies focused on the Maldives that address current challenges and provide actionable insights. Therefore, to address this, efforts to promote voluntary blood donation and educate the public about its importance are vital.

This study is aimed at conducting a qualitative analysis to assess voluntary blood donors′ knowledge, attitudes, and motivations in the Maldives. Through one‐on‐one interviews, it seeks to identify factors influencing blood donation and recommend strategies to increase donor participation. The findings will assist blood banks and health authorities in refining donor engagement and retention approaches.

## 2. Methodology

The study employed a qualitative descriptive approach to explore participants′ experiences about motivational factors and obstacles to blood donation. Additionally, this study seeks donors′ opinions on motivating, recruiting, and retaining donors to create a safe donor pool, enabling the interviewer to understand why potential donors refrain from donating blood.

A qualitative approach was considered appropriate, as it allows in‐depth exploration of blood donors′ perspectives on the blood donation process at a tertiary care hospital in the Maldives. This is the only government tertiary care hospital and plays a significant role as a major blood bank in the Maldives.

A purposive sampling technique was employed to recruit participants who had direct experience relevant to the study and were able to provide rich information. A total of 12 participants were selected for the study. The study included voluntary donors who registered and had donated during the study period (March 2015–March 2017). This sample size was deemed appropriate for a qualitative inquiry, as qualitative research is more focused on analytical depth and contextual understanding. Data collection was guided by the principle of data saturation, and by the tenth interview, no new themes emerged, and subsequent interviews yielded repetitive information, confirming the sample size was sufficient to capture the key issues for this study. The sample size was considered adequate, as the main objective was to explore experiences in depth rather than to achieve statistical generalization [14, 15].

Donors were selected among the population donors who visited the tertiary care hospital blood bank to donate blood, considering the number of blood donations. The purpose was to understand the factors that motivate both groups, find obstacles and overcome them, and, through the donor′s point of view, find ways to retain them.

• First‐time donors—four donors
• Donors who had donated 3–4 times during the time frame—four donors
• Donors who had donated at least 3 times each year (were considered regular donors)—four donors

The questions were designed to gather as much information as possible from the participants regarding their knowledge, attitudes, and motivations toward voluntary contributions, as well as their perceptions of the existing donation process. The questionnaire (Appendix 1) was validated by three key experts in blood donor recruitment and retention, who also had expertise in qualitative research and public health practice.

The experts evaluated the interview questions for relevance of the research objectives, clarity, and appropriateness for the study population.

All participants who accepted the research invitation provided consent before the interview, which explained the study and confidentiality details.

Data was collected through semistructured interviews conducted by the primary researcher, all of which were audio‐recorded and transcribed. Data analysis was done using thematic analysis. The participants were asked to counter‐check whether any information was missing and whether the interviewer understood the information as they had expressed it.

The interviews were transcribed on a Microsoft Word sheet, and the audio recordings were listened to for data development. The transcripts were analyzed and read thoroughly, and coding was done.

Ethical approval was from the Institute for Research and Innovation (2017/E‐147), Tertiary Care Hospital and National Health Research Committee.

## 3. Results

All participants were 18 years or older. The study included both male and female donors with varying donation frequencies, including first‐time donors, donors who had donated three times, and regular donors as shown in Table 1. This distribution enabled exploration of knowledge, attitudes, and motivational factors across different levels of donation experience.

**Table 1:** Participant characteristics (*n* = 12).

Variable	Category	*n*
Age	≥ 18 years	12
Gender	Male	9
	Female	3
Donation frequency	Donated once	4
	Donated 3 times	4
	Regular donors	4

**Figure 1:**
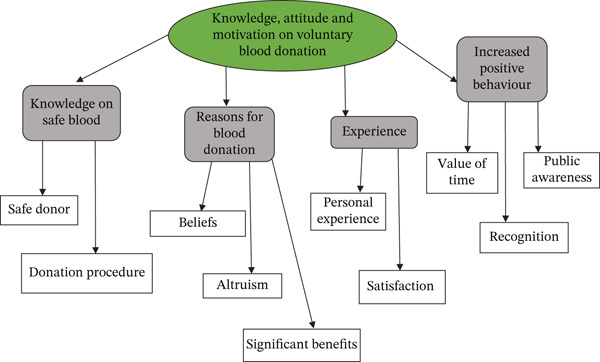
The generated themes with subthemes.

The data analysis framework guided the analytic process of this research. The transcribed interviews revealed the knowledge, motivation, and attitudes toward blood donation among voluntary donors. As a result, this research explored the donors′ perspectives and identified the shared and differing points of view. Four main themes were generated, along with 10 subthemes as shown in Figure 1. The study period was from March 2015 to March 2017, and it was done among donors who had donated once, donors who had donated blood three times, and donors who had donated regularly. Four donors from each group were approached to understand the factors associated with knowledge, attitude, and motivation for blood donation.

Almost all the participants were donating blood for selfless reasons. However, the knowledge of blood donation varied. Except for two individuals, no other has had an adverse reaction to blood donation, and only one individual has developed a negative attitude due to the reaction. All the participants had given their views and recommendations on the present donation process and how to recruit and retain blood donors. All agreed that a sufficient blood supply is needed, and it would only be possible by regular blood donations.

Based on these, four main themes were identified: knowledge of safe blood, reasons for blood donation, experience, and increased positive behavior. Ten subthemes were identified as follows.

### 3.1. Knowledge of Safe Blood

Most of the participants agreed that the safest donor is a voluntary donor, reflecting an understanding of the importance of donor motivation and screening ensuring blood safety Further, they explained that they donate blood regularly; hence, their blood gets tested every 3–4 months.

*“Voluntary donors give blood regularly 3-4 times a year and every time their blood is tested. And since we are voluntary donors, we try to be ready for the next donation and try not to do things that can differ us.”* (Respondent 01)

Of the 12 participants, three believed that a directed family donor was the safest.

*“The safest donor is a family member or someone you know. You know who that person is, his behaviour, and family members have close blood bonds.”* (Respondent 06)

### 3.2. Donation Procedure

The participants′ knowledge of blood donation and donor acceptance criteria was good, particularly the requirement that donors need to be healthy, disease‐free, and adults aged 18 years and above. This awareness suggests that the donors do have the adequate basic understanding of safety principles. However, uncertainty was raised with the volume of blood withdrawn indicating a knowledge gap. Out of the 12 participants, seven were able to provide the exact volume in a unit of blood; others did not know or had not given it much thought.

*“One pint of blood never gave a thought to it; the nurse did tell me once but never gave importance to it. I think it is 400 or 450.”*

(Respondent 01)

*“One pint of blood, I do not know. never thought to ask or find out.”*

(Respondents 06 and 08)

The development of a positive attitude toward blood donation plays a vital role in sustaining a sufficient blood supply. A positive attitude is also an important motivational factor influencing blood donors to donate regularly.

### 3.3. Beliefs

Beliefs are crucial in changing a person′s attitude toward blood donation. There are many misconceptions about blood donation; however, none of the donors who participated harbored any. Although two respondents reported having heard community narratives linking to donation weight gain, they did not accept these claims as valid. These findings suggest a capacity to critically evaluate misinformation, likely supported by prior donation experience and adequate knowledge.

*“Some people say that you get fat after donating, but that doesn′t happen. After donation I feel like eating more, but I think it′s all psychological.”* (Respondent 03)

The respondents believed that they were doing some community work through blood donations and that their continued blood donation would keep a sufficient blood supply in the blood bank.

*“My reasons for blood donation is purely for community service, I do not do it for any personnel benefit. There will be blood in the blood bank, available for the people when they need it.”* (Respondent 01)

One participant responded that

*“I donate for the community, it is my contribution for a sufficient blood supply and after donation I feel like I have contributed to the community and gives me mental peace.”* (Respondent 02)

### 3.4. Altruism

All participants reported that their primary motivation of donating blood was to help individuals in need of transfusions and to contribute to maintaining an adequate blood supply. This motivation reflects strong altruistic values and sense of social responsibility, which are two main key drives for voluntary blood donation.

*“There are so many Thalassemia children and kidney disease people who need blood. And many times, I see people calling friends and announcing over the television for blood. So, I think if it is available it would be a bit easy for them in getting blood.”* (Respondent 10)

Continuous donation was in a way to ease the patient′s family from struggling to find. It was a way of reaching out to those in need.

*“The patient′s family doesn′t have to struggle to find blood or blood donors. There will always be blood available for a person in need.”* (Respondent 07)

### 3.5. Significant Benefits

Although the participants primarily viewed blood donation as a way of helping out without any personal benefits, many shared the understanding that it provided significant health benefits. This statement reflects the recognition of health benefits can enhance perceived benefits while maintaining a positive attitude toward blood donation.

*“We will have fresh blood circulating in our system and I think health wise you get a regular check-up and you will get to know if your blood pressure rises.”* (Respondent 06)

Another respondent had stated:

*“Scientifically blood donation is good for cardiovascular problems and for people with lipid problems like dyslipidaemia.”* (Respondent 02)

One participant believed that regular blood donation might negatively impact health, which could reduce donor participation in regular donations.

*“Continuous regular donations can make my haemoglobin levels low.”* (Respondent 03)

### 3.6. Personal Experience

All the participants expressed satisfaction toward the donation process. Positive interpersonal experiences can enhance perceived behavioral control by reducing anxiety and uncertainty. However, for two participants, it was eventful.

*“I had come for my fifth donation, and the nurse couldn′t find a vein. She said it happens sometimes and was poking the needle trying to find the vein. Then she wanted to check the other hand and the same thing happened. I imagine, she pricked me on both hands and couldn′t find the vein and then told me to come after a week. I was very disappointed but I did come after one week and donated with one try. Both my hands were blue due to the pricking, and the sisters did apologize. The experience didn′t have any negative effect on my willingness. I just passed it as a bad day.”* (Respondent 01)

Some participants also started donating because they had experienced how difficult it was to find donors and how important it was to have access to blood when it was needed.

*“My mother had an ectopic pregnancy and she was in need of blood, a lot of my friends came to donate. My mother went into a hypovolemic shock. During that period, I also got my blood checked and donated. The experience was like a motivation for me, and that′s the reason I regularly donate.”* (Respondent 02)

### 3.7. Satisfaction

Ensuring donor satisfaction is essential for encouraging repeat visits to the blood bank, as a positive experience directly influence donor willingness to continue donating.

*“I was happy and at ease. Everything went well, I donated. It was a bit painful but the staff were very nice. As it was my first time they very observant and I got some free medical advice too.”* (Respondent 08)

### 3.8. Increasing Positive Behavior

Finding ways to motivate and attract the community to voluntary blood donation is important. The present donation process of the blood bank was faultless; however, there were many areas where it could be improved.

### 3.9. Value of Time

Participants consistently emphasized the value of time, noting that the blood donation process was perceived as time‐consuming. This highly reflects a practical barrier that may influence donation behavior.

“*The present donation process is ok but it′s a bit time consuming. The last time I came I had to wait about 45 mins as an emergency had come and some donors had to be bled, and since there is only one chair available, one by one had to be bled.*” (Respondent 04)

### 3.10. Recognition

The study observed that almost all the participants agreed on recognizing the blood donor in some form. This would be a small token of recognition that would motivate and encourage the present donors and attract new donors as well.

*“A token or a badge can be given, like after the third donation. This is not to bribe them or giving them a gift, but as a token of recognition. Appreciation is good, so when people ask them what the badge is for they will say its after the third donation. It will motivate others also to donate and like this you can increase donors and get new donors.”* (Respondent 02 & 05)

One respondent had a different point of view.

*“I don′t think we should give any gift or token.it is something done for a good cause, I don′t think any reward is needed.”* (Respondent 08 & 09)

### 3.11. Public Awareness

The participants believed that to sustain any event, it is important to inform the public about its importance. In the same manner, it is important for the public to be aware of procedures and to know about safe blood.

*“Encouragements can be done through advertisements, I think people have to know that they can donate and that it is safe. In the same manner, make them aware on the concept of safe blood.”* (Respondents 11 and 12)

## 4. Discussion

Blood donation plays a vital role in healthcare systems, meeting the blood and blood products required for various medical conditions, including trauma, surgeries, and chronic diseases. In the Maldives, the need for blood is exceptionally high due to the prevalence of thalassemia, as highlighted in the Maldives Health Profile 2016. Thalassemia patients rely heavily on blood transfusions, yet hospital blood banks predominantly depend on directed blood donations (70%), with only 30% coming from VNRDs [11, 15].

Studies by WHO emphasize that blood donations from VNRDs are essential to ensuring safe and high‐quality blood products [16]. However, like many countries, the Maldives faces challenges in maintaining sufficient and safe blood supplies. Research has shown that recruiting low‐risk blood donors remains a global challenge in developed and developing countries [5]. To address these issues effectively, donor recruitment and retention programs must focus on understanding donors′ knowledge, attitudes, and motivations, as this information can guide the development of tailored strategies and campaigns to encourage more regular voluntary blood donors [11].

### 4.1. Knowledge

Knowledge about blood donation is a crucial factor in encouraging voluntary donations. It helps dispel fear, correct misconceptions, and foster a positive attitude toward donation. According to [17], knowledge is a prerequisite for timely and effective voluntary blood donation. This study assessed the knowledge levels of 12 voluntary blood donors registered at a tertiary care hospital and found that, while most donors had a general understanding of blood donation, there were notable gaps. For instance, 25% of participants believed that family donors were the safest, reflecting a misconception that could hinder voluntary donation. However, 75% correctly identified voluntary donors as the safest and understood the rationale. Addressing these gaps through targeted awareness campaigns can further enhance donor knowledge and trust in blood donation.

### 4.2. Attitude Toward Blood Donation

Personal experiences, beliefs, and perceptions of the donation process shape attitudes toward blood donation. Positive attitudes are crucial for motivating donors to continue donating. Studies in 16 countries have shown that misconceptions, fear, and distrust in the system can lead to negative attitudes [18, 19], while awareness and knowledge can foster positivity [10, 17]. In this study, all participants exhibited positive attitudes toward blood donation, with many citing social responsibility and altruism as key motivations. Additionally, the quality of care and friendly interactions with staff significantly reinforced positive experiences. Ensuring a seamless and welcoming donation process is vital to maintaining these attitudes and encouraging repeat donations.

In this study, participants consistently demonstrated positive attitudes toward blood donation, driven primarily by social responsibility and altruistic values. These findings align with altruism theory, which posits that prosocial behavior is motivated by concern for others rather than personal gain. Additionally, the results can be interpreted through the theory of planned behavior (TPB), which holds that positive attitudes toward the act of donation, reinforced by favorable experiences, increase behavioral intention and the likelihood of repeat donation. Supportive, friendly interactions with healthcare staff further contribute to positive donor experiences, enhancing perceived behavioral control by reducing anxiety and uncertainty associated with the donation process. Collectively, these factors underscore the importance of a seamless, respectful, and welcoming donation environment in sustaining positive donor attitudes and promoting repeat voluntary blood donation.

In the Maldivian sociocultural context, strong communal ties, family influence, and collective responsibility play a significant role in shaping health‐related behaviors, including blood donation. The emphasis on community wellbeing and mutual support fosters altruistic motivations, particularly in emergency or healthcare settings where blood donation is viewed as a moral and social obligation. Additionally, trust in healthcare providers and institutions is highly valued, and positive interpersonal interactions with donation staff can significantly influence donor willingness and retention. However, traditional beliefs and shared community narratives may also perpetuate misconceptions about blood donation, highlighting the need for culturally sensitive education and awareness initiatives. Addressing these sociocultural factors through community‐based engagement and targeted communication strategies may enhance donor participation and sustain voluntary blood donation in the Maldivian context, where a high number of thalassemia patients are in need of blood.

### 4.3. Motivating Donors for Recruitment and Retention

Blood banks must recruit safe and low‐risk blood donors to maintain a sufficient blood supply. To achieve this, they must strive to develop positive community attitudes to voluntary blood donation to motivate, recruit, and retain sufficient safe blood donors [5]. A study done in Tehran highlights that the recruitment process requires constant efforts and active work and the importance of recruiting new volunteers from the community [12].

Many factors play an important role in recruitment and retention. Knowledge, proper awareness programs, and motivation are key factors that influence the recruitment and retention of voluntary blood donors.

### 4.4. Limitations of the Study

One key limitation of this study was its sample size of only 12 participants. This small sample may not adequately represent blood donors′ diverse perspectives and experiences across the Maldives. A more extensive and varied sample could provide more generalizable findings and reflect the broader population. Another limitation was the study′s geographical focus, which was restricted to voluntary blood donors registered at the tertiary care hospital blood bank. This scope may not capture the challenges, attitudes, and practices in other blood banks, where blood donation practices might differ.

The reliance on self‐reported data posed a potential risk of social desirability bias, as participants might have provided responses they deemed favorable or socially acceptable rather than being entirely truthful. This could have affected the accuracy of the data collected on donor knowledge, attitudes, and motivations.

Additionally, the study lacked a longitudinal analysis, capturing data at a single point in time. This approach made examining changes in donor knowledge, attitudes, and motivations difficult over time, which could have provided a deeper understanding of factors influencing repeated blood donation. Furthermore, the study provided limited exploration of external influences, such as cultural norms, societal beliefs, or public health campaigns, which could play a significant role in shaping donor behavior and attitudes. A more comprehensive analysis of these factors would have added depth to the findings.

Finally, while the study relied on qualitative data to gather in‐depth insights, the absence of quantitative measures limited the ability to statistically validate findings or assess the prevalence of specific knowledge gaps and attitudes. Combining qualitative and quantitative methods could strengthen future research by providing rich narratives and measurable evidence.

Addressing these limitations in future studies can lead to a more comprehensive understanding of blood donation practices and donor behaviors in the Maldives, ultimately helping to develop targeted strategies for improving voluntary blood donation rates.

### 4.5. Implications for Policy, Institutions, and Individuals

#### 4.5.1. Policy Implications

The findings of this study highlight the urgent need for robust policy interventions to improve voluntary blood donation rates in the Maldives. Central to this is establishing a centrally coordinated blood banking system, which would ensure the standardization of practices across all blood banks, promote equitable access to safe and sufficient blood supplies, and enable efficient management of blood collection, testing, storage, and distribution. Such a system would also enhance crisis management by enabling the rapid redistribution of blood during emergencies. Policies should further prioritize awareness campaigns to educate the public on the importance of voluntary blood donation, dispel misconceptions, and foster trust in the system. The National Blood Policy should emphasize the creation of mandatory recruitment and retention programs targeting young donors and underserved communities. Additionally, frameworks to monitor and evaluate the effectiveness of donor engagement initiatives are essential for ensuring sustainable improvements.

#### 4.5.2. Institutional Implications

At the institutional level, blood banks and healthcare facilities must play a proactive role in enhancing the donor experience and building trust. Institutions should focus on streamlining the donation process, such as reducing waiting times by adding donor chairs and improving infrastructure in waiting areas. Investing in staff training is critical to fostering a donor‐friendly environment and ensuring high‐quality care. Institutions should also leverage data‐driven approaches to track donor behavior, identify trends, and engage donors effectively. Partnerships with schools, workplaces, and community organizations can expand outreach and attract new donors. Regular feedback mechanisms should also be implemented to address donor concerns and improve retention.

#### 4.5.3. Individual Implications

On an individual level, the study underscores the importance of addressing personal barriers to blood donation, such as fear, misconceptions, and lack of motivation. Educational initiatives should focus on dispelling myths, highlighting the process′s safety, and emphasizing donors′ critical role in saving lives. Recognizing and appreciating individual contributions can significantly motivate repeat donations. Strategies such as personalized donor recognition through certificates, appreciation tokens, and milestone awards combined with celebrations on significant occasions like World Blood Donor′s Day can create a sense of belonging and appreciation among donors. These initiatives and targeted reminders and acknowledgements on special days can strengthen donor loyalty, enhance engagement, and encourage long‐term commitment to blood donation efforts. Additionally, promoting a sense of altruism and social responsibility through relatable messaging can inspire individuals to view blood donation as a valuable and impactful contribution to their communities.

## 5. Conclusion

The study underscored the need to increase the number of regular voluntary blood donors by implementing awareness campaigns and prioritizing targeted recruitment and retention strategies. While all participants expressed positive attitudes toward blood donation, certain factors could foster negative perceptions over time if left unaddressed. Consequently, integrating the recommendations provided by donors into policy and practice could help enhance strategies, ensuring better outcomes in the future.

Enhancing any service requires adopting its users′ perspective. Likewise, promoting regular voluntary blood donation necessitates listening to and acting upon donors′ views. The voluntary donors in this study offered valuable insights and recommendations to boost donor motivation and improve blood donation rates. Recognizing time as a vital resource, it is essential to act swiftly on these insights to achieve meaningful and lasting improvements.

## Author Contributions

**Khadeeja:** conceptualization, methodology, data curation, writing. **Aishath Selna:** conceptualization, writing – review and editing. **Adam Khaleel Yoosuf:** proofreading and editing.

## Funding

No funding was received for this manuscript.

## Conflicts of Interest

The authors declare no conflicts of interest.

## Data Availability

The data that support the findings of this study are available on request from the corresponding author. The data are not publicly available due to privacy or ethical restrictions.

## Appendix 1: Interview Guide

### Interview Guide (one on one)

The questionnaire is a part of a research I am doing to find out the knowledge, barriers and motivations towards voluntary blood donation. This is needed as part of my dissertation on the topic. It would be appreciated if you would participate and answer the questions truthfully and to the best of your knowledge.

Please tick {√} where appropriate

1. Can you tell me your age?

2. Gender

3. Can you tell me your educational level and profession/designation?

4. Do you know your Blood Group? …………………………..

5. Have you ever donated blood? ………………

If yes,

How many times? ………………………….

6. Opening question

a. What can you tell me about your experience of donating blood?

b. What was the reason for your first donation?

c. What can you tell me about your reasons for regularly donating blood?

7. Knowledge

9.1 Do you know

a) How old you have to be to donate blood? …………………………………

b) How much you should weigh? ……………………………………

c) How much volume is one unit of blood? ………………………………..

d) Who is the safest donor?

1. Family member

2. Replacement Donor

3. Voluntary Donor

Why:

…………………………………………………………………………………………………

……………………………..

e) How many times can you donate blood in a year?

…………………………………….

8. Attitude

10.1 What are your reasons for donating blood?

10.2 For you, what are the personal benefits associated with regularly donating blood?

10.3 What benefits does your continued donation provide to the community?

10.4 What do you believe are the disadvantages of regularly donating blood?

10.5 What are your feelings after a blood donation?

11 Previous donation experience

11.1 How has your level of satisfaction with previous donation experiences impacted on your donation behaviour?

…………………………………………………………………………………………………

…………………………………………………………………………………………………

………

11.2 Have you ever had any bad physical reactions to donating?

…………………………………………………………………………………………………

……………………………………………………………………………………………

…………………………………………………………………………………………

(If yes) What has been the impact of these negative physical reactions on your willingness to donate blood again?

…………………………………………………………………………………………………

……………………………………………………………………………………………

………………………………………………………………………………………

12. What is your opinion on the present donation procedure?

…………………………………………………………………………………………………

……………………………………………………………………………………………

……………………………………………………………………………………………

………..

13. From your point of what can be done to further improve the blood donation service?

…………………………………………………………………………………………………

…………………………………………………………………………………………………

………………………………………………………………………………………………

Thank you for your precious time and participation in the survey.

## References

[1] Myers D. J. and Collins R. A. , Collins J. , Blood Donation, Continuing Education Activity, 2024, StatPearls Publishing LLC.

[2] Bajwa H. and Basit H. , Continuing Education Activity, Thalassemia, 2023, StatPearls Publishing LLC.

[3] Roh J. , Park S. , and Kang H. J. , Recent Trends in Perioperative Blood Transfusion During Elective Kidney Transplantation, Korean Journal of Transplantation. (2023) 37, no. 3, 197–202, 10.4285/kjt.23.0041, 37751967. 37751967 PMC10583966

[4] Tebabal B. , Anagaw T. F. , Adamu A. , and Atnafu D. D. , Factors Influencing Blood Donation Practice Among Health Care Providers of Public Hospitals in Bahir Dar City, North West Ethiopia: A Case Control Study, Journal of Blood Medicine. (2023) 14, 487–498, 10.2147/JBM.S423013, 37674760. 37674760 PMC10479530

[5] Alanazi A. E. , Almulla B. F. , Alanazi S. S. , Alshammari S. M. , Aldossary A. A. , Alanazi S. M. , Alanazi T. B. , Almulla B. R. , Alanazi S. M. , and Alshammari S. K. , Knowledge and Barriers About Blood Donation and Associated Factors in Saudi Arabia: A Systematic Review, Cureus. (2023) 15, no. 11, 1–8, 10.7759/cureus.48506. PMC10706263 38074024

[6] Nagurney A. and Dutta P. , Competition for Blood Donations, Omega. (2019) 85, 103–114, 10.1016/j.omega.2018.06.001, 2-s2.0-85048769451.

[7] WHO , Universal Access to Safe Blood Transfusion, 2008, World Health Organisation.

[8] World Health Organization , Blood Safety and Availability, 2023, World Health Organization.

[9] Planning and International Health Division , Maldives Health Profile 2016, 2016, Ministry of Health, Republic of Maldives.

[10] Waheed F. , Fisher C. , Awofeso A. , and Stanley D. , Carrier Screening for Beta-Thalassemia in the Maldives: Perceptions of Parents of Affected Children Who Did Not Take Part in Screening and Its Consequences, Journal of Community Genetics. (2016) 7, no. 3, 243–253, 10.1007/s12687-016-0273-5, 2-s2.0-84978083258, 27393346. 27393346 PMC4960032

[11] Angastiniotis D. , The Maldives WHO Mission August 2014, Thalassaemia International Federation, 2014, WHO.

[12] Toogeh G. , Mirrezaie S. M. , Tabatabaee S. M. , Saber H. R. , Assari S. H. , and Shariati M. , Women′s Blood Donation: A Qualitative Study Exploring the Reasons for Non-Donation of Blood in Female Staff at Tehran Blood Transfusion Center, International Journal of Health Studies. (2015) 1, no. 3, 24–28.

[13] Ministry of Health , National Guideline on Clinical Use of Blood, 2022, Ministry of Health.

[14] Marshall M. N. , Sampling for Qualitative Research, Family Practice. (1996) 13, no. 6, 522–525, 9023528, 10.1093/fampra/13.6.522, 2-s2.0-0030453840. 9023528

[15] Creswell J. W. , Qualitative Inquiry and Research Design: Choosing Among Five Approaches, 2012, 3rd edition, SAGE Publications.

[16] WHO Expert group , Expert Consensus Statement on achieving self-sufficiency in safe blood and blood products, based on voluntary non-remunerated blood donation (VNRBD), Vox Sanguinis. (2012) 103, no. 4, 337–342, 10.1111/j.1423-0410.2012.01630.x, 2-s2.0-84867855845. 22690746

[17] World Health Organization , Voluntary non-Remunerated Blood Donations to Ensure Blood Safety in the WHO South-East Asia Region to Support Universal Health Coverage, 2023, World Health Organization South East Asia.

[18] Jemberu Y. A. , Esmael A. , and Ahmed K. Y. , Knowledge, Attitude and Practice Towards Blood Odnation and Associated Factors Among Adults in Debre Markos Town, Northwest Ethiopia, 2016, 16, no. 23, BMC Hematology. 10.1186/s12878-016-0062-8 PMC5011944 27602227

[19] Eltewacy N. K. , Ali T. H. , Owasis T. A. , Alkanj S. , and Ebada M. , Unveiling Blood Donation Knowledge, Attitude, and Practices Among 12, 606 University Students: A Cross-Sectional Study Across 16 Countries, Scientific Reports. (2024) 14, no. 1, 1–16, 10.1038/s41598-024-58284-4, 38589387. 38589387 PMC11001850

